# Early in-hospital discontinuation of aspirin on the first post-procedural day after percutaneous coronary stent implantation in patients on direct oral anticoagulation

**DOI:** 10.3389/fcvm.2023.1265452

**Published:** 2023-12-13

**Authors:** Philipp von Stein, Lukas Seitz, Hendrik Wienemann, Christopher Hohmann, Till Baar, Stephan Baldus, Marcel Halbach

**Affiliations:** ^1^ Clinic III for Internal Medicine, Faculty of Medicine and University Hospital Cologne, University of Cologne, Cologne, Germany; ^2^ Faculty of Medicine, Institute of Medical Statistics and Computational Biology, University of Cologne, Cologne, Germany

**Keywords:** PCI, aspirin, DOAC, triple therapy, dual therapy

## Abstract

**Background:**

Previous trials investigating antithrombotic therapy with a direct oral anticoagulant (DOAC) and a P2Y12 inhibitor after percutaneous coronary intervention (PCI), termed dual therapy, allowed a short period of triple therapy including a DOAC, a P2Y12 inhibitor, and aspirin.

**Aims:**

This study aimed to determine whether discontinuation of aspirin on the first post-procedural day is safe or causes ischemic events.

**Methods:**

Ischemic and bleeding events during hospitalization were investigated retrospectively in all patients treated with dual therapy (DOAC + P2Y_12_ inhibitor, designated as group 1) or triple therapy (DOAC + P2Y_12_ inhibitor+aspirin, designated as group 2) from day 1 after PCI at our center.

**Results:**

Of 4,564 consecutive PCI procedures, 1,059 (23.2%) had an indication for OAC. Of these, 322 met the inclusion criteria for group 1 and 62 for group 2. Baseline characteristics, CHA_2_DS_2_-VASc and HAS-BLED scores showed no relevant differences between the two groups, and the main indication for DOAC therapy was atrial fibrillation in both groups. Approximately ¼ of patients were treated for acute coronary syndrome. The mean length of post-procedural hospitalization was 2.1 ± 2.5 and 2.2 ± 3.0 days in group 1 and 2, respectively (*p* = 0.305). One patient per group suffered a TIA (*p* = 0.297). There were no other ischemic events and no statistically significant differences in bleeding events. A subgroup analysis of cases hospitalized for ≥2 post-procedural days (group 1: 100 cases, mean 4.4 ± 3.4 days vs. group 2: 25 cases, mean 4.0 ± 4.1 days) confirmed these results.

**Conclusion:**

The initiation of dual therapy and thus discontinuation of aspirin on the first postprocedural day appears to be safe with respect to short-term ischemic events in a real-world population. Almost ¼ of patients undergoing PCI have an indication for OAC, highlighting the relevance of this issue.

## GRAPHICAL ABSTRACT

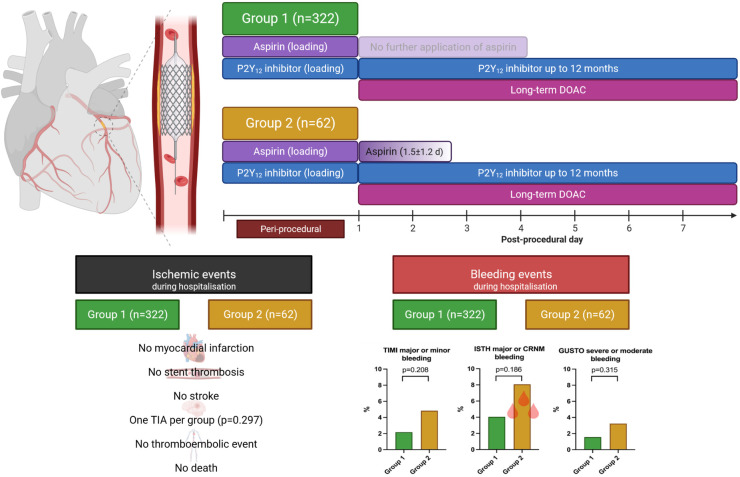

To assess whether discontinuation of aspirin on the first post-procedural is safe or leads to ischemic events, we studied patients who received either dual therapy (DOAC + P2Y12 inhibitor, referred to as group 1) or triple therapy (DOAC + P2Y12 inhibitor+aspirin for ≥1-day, referred to as group 2) throughout their hospitalization. One patient per group suffered a TIA, and both patients were discharged one day after the procedure. No other ischemic events occurred. Patients in group 2 had numerically more bleeding events, but these differences were not significant. DOAC, direct oral anticoagulation; GUSTO, Global Use of Strategies to Open Occluded Arteries; ISTH, International Society on Thrombosis and Haemostasis; TIA, transient ischemic attack; TIMI, Thrombolysis in Myocardial Infarction.

## Introduction

Approximately 5%–10% of patients undergoing percutaneous coronary intervention (PCI) have an indication for long-term oral anticoagulation (OAC), most commonly to prevent cardiac thromboembolism in patients with atrial fibrillation (1–3). In addition, patients undergoing PCI require dual antiplatelet therapy (DAPT), consisting of aspirin and a P2Y_12_ inhibitor, to prevent coronary thrombotic complications (4, 5). The combination of OAC with DAPT, so called triple therapy, is associated with a high rate of bleeding events (3, 6). Randomized controlled trials have shown a reduction in bleeding events, without significant differences in ischemic events, with dual therapy, which combines OAC with a P2Y_12_ inhibitor instead of triple therapy (7–11). In these trials, periprocedural aspirin therapy was mandatory, so it can be assumed that all patients received at least a short course of triple therapy, most likely until randomization (12).

Based on the current evidence, recent European guidelines and a 2021 North American consensus document recommend dual therapy as mid-term strategy, but triple therapy for up to one week for most patients (up to 30 days in patients with high ischemic risk) (4, 5, 12, 13). The guidelines do not recommend a more precise duration within this 1-week period because of a lack of evidence. Therefore, we investigated whether the exclusive procedural application of aspirin and the very early discontinuation of aspirin from the first post-procedural day is safe or may cause ischemic events.

## Methods

### Study population and data collection

In this retrospective, single-center study, the post-procedural antithrombotic therapy of all patients who underwent PCI with stenting at the Heart Center of the University Hospital of Cologne between January 2017 and December 2020 was evaluated and assessed for the indication of long-term OAC to determine how frequently the dual or triple therapy situation occurs in a real-world population. All patients with an indication for long-term OAC who were treated with direct OAC (DOAC) were included in the present analysis and divided into two groups: Group 1 included patients treated with dual therapy consisting of a DOAC and the P2Y_12_ inhibitor clopidogrel from the first post-procedural day, thus patients in group 1 received aspirin only during the procedure or before the procedure, e.g., in case of an acute coronary syndrome (ACS). Group 2 included patients treated with triple therapy (DOAC, the P2Y_12_ inhibitor clopidogrel, and aspirin) from the first post-procedural day and for ≥1 day. If patients received more than one PCI with stenting during the study period, all PCIs were included separately in the analysis considering that a subsequent PCI is associated with an additional risk of ischemic or bleeding events. However, for simplicity, we refer to cases/PCIs as patients throughout the manuscript and provide a detailed description in Supplementary Table S1.

Patients who were treated with low-molecular-weight heparin, unfractionated heparin, vitamin K antagonists, low-dose DOAC, or DAPT alone until discharge were excluded. Furthermore, we excluded patients who had cardiogenic shock, did not receive stenting, had contraindications to anticoagulation, aspirin or any P2Y_12_ inhibitor, did not have a blood draw on admission, had insufficient documentation in the medical records or were receiving a P2Y_12_ inhibitor other than clopidogrel from the first day after the intervention (loading with another P2Y_12_ inhibitor and switching to clopidogrel on the first post-procedural day was allowed).

The data collection was performed retrospectively with approval of the local ethics committee at our academic center (No. 22-1394-retro). Data were collected based on the local hospital database, including cardiac catheterization protocols, laboratory values, patient charts, and discharge letters. Follow-up included the post-procedural period in the hospital. In addition, a subgroup analysis of patients that were hospitalized for ≥2 days was performed.

### Baseline and procedural characteristics

At baseline, established risk factors for stent thrombosis, such as age, diabetes mellitus, type of presentation (NSTEMI/STEMI), prior myocardial infarction, smoking status, platelet count, anemia, end-stage renal disease, and prior stent thrombosis were assessed (14). Furthermore, stroke risk was calculated using the CHA_2_DS_2_-VASc score and bleeding risk was calculated using the HAS-BLED score at baseline (15, 16). In addition, any pre-existing antiplatelet therapy was recorded.

Assessed lesion-related parameters that were known to be associated with stent thrombosis included multivessel disease, diseased vessel (i.e., left anterior descending artery [LAD], left circumflex artery [LCX], right coronary artery [RCA], and bypass graft), and bifurcation lesion (14). Collected procedure-related parameters were also associated with an increased rate of stent thrombosis, such as type of stent (drug eluting stent [DES] and bare metal stent [BMS]), number of stents implanted, stent length, stent diameter of the smallest implanted stent, bifurcation stenting, overlapping stents, stented vessel, use of glycoprotein IIb/IIIa inhibitors, and missing heparin pretreatment (14).

### Post-procedural antithrombotic therapy

Post-procedural antithrombotic therapy administered from the first post-procedural day was recorded (i.e., aspirin, clopidogrel, apixaban, dabigatran, edoxaban, and rivaroxaban). Based on these data, patients were divided into groups 1 and 2 (see above).

### Endpoints

All clinical endpoints were collected as part of standard clinical care. Ischemic events included myocardial infarction, stent thrombosis, stroke, transient ischemic attack (TIA), and any other thromboembolic event during hospitalization. Bleeding events during hospitalization were determined based on the following established definitions: GUSTO (Global Use of Strategies to Open Occluded Arteries), ISTH (International Society on Thrombosis and Haemostasis), and TIMI (Thrombolysis in Myocardial Infarction) (17–21). These definitions are provided in Supplementary Tables S2–S4.

### Statistical analysis

Categorical variables are reported as frequencies and percentages, *n* (%). Continuous variables are presented as mean ± standard deviation (SD) or median with interquartile range (IQR), as appropriate. Distribution of continuous variables was assessed with the Kolmogorov-Smirnov test. Comparisons between groups were performed with the Fisher's exact test for categorical variables and the Student's *t*-test or Mann–Whitney *U*-test for continuous variables, depending on data distribution. Two-sided *p*-values <0.05 were considered statistically significant. Data analysis was performed with IBM SPSS Statistics version 28.0 (IBM, Armonk, NY, USA) and Excel (Microsoft Corporation, Redmond, WA, USA). Graphs were created with Prism version 8.4.0 (GraphPad Software Inc., San Diego, CA, USA) and the BioRender.com platform.

## Results

### Study population and baseline characteristics

**Figure 1:**
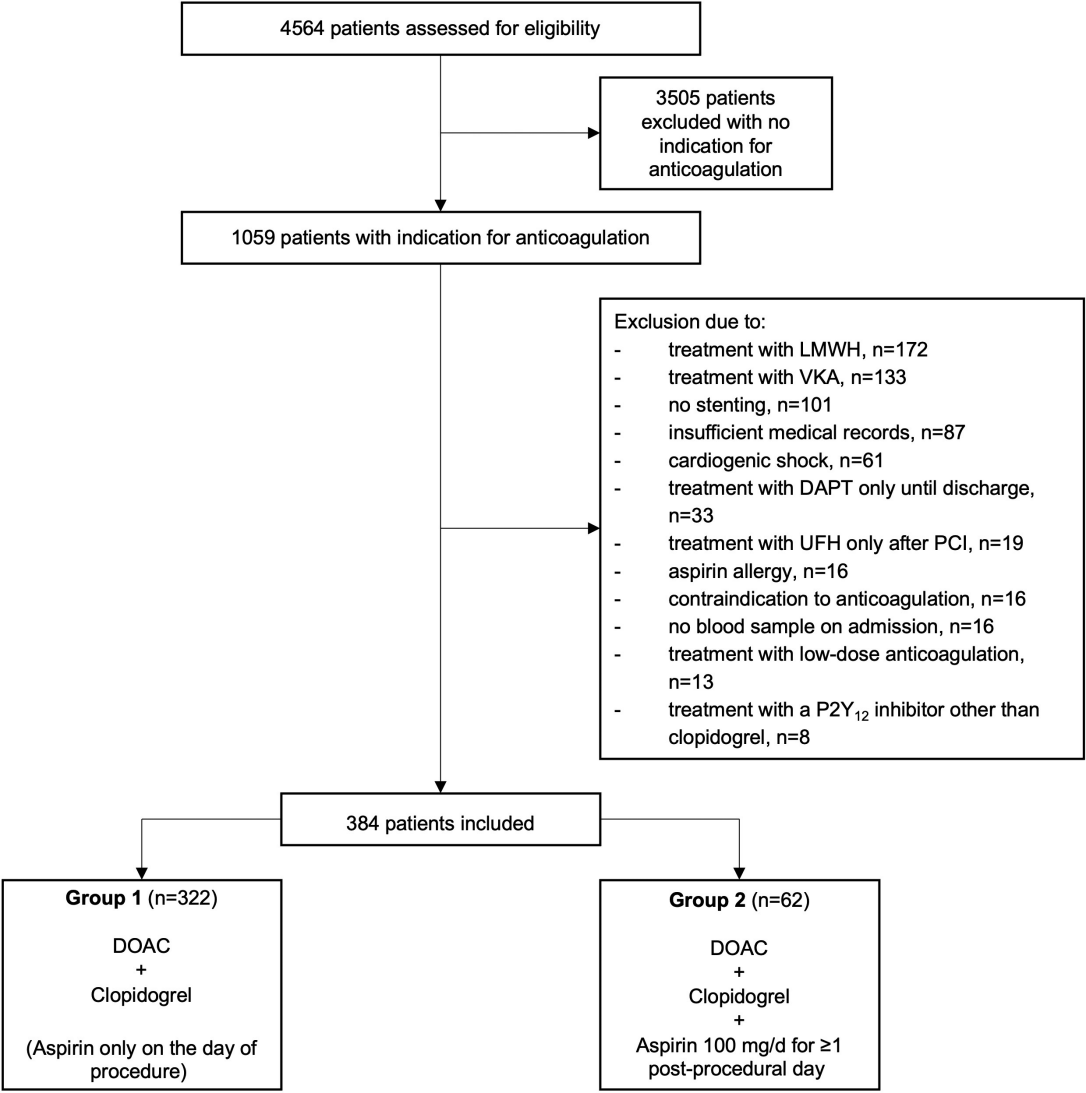
Patient disposition.

During the study period, a total of 4,564 patients underwent PCI at our center, of whom 1,059 (23.2%) had an indication for anticoagulation. Of these, 384 complied with all criteria to be included in the present study. 322 patients were treated with dual therapy from the first post-procedural day and included in group 1, and 62 patients were treated with triple therapy from the first postprocedural day and assigned to group 2 (Figure 1, Supplementary Table S1). Baseline characteristics of the two groups are provided in Table 1. The median age across groups was 77.6 (70.4–81.7) years, 27.3% were female. The incidence of drug-treated hypertension, diabetes, heart failure, previous stroke, TIA and thromboembolism was comparable between the two groups, resulting in similar CHA_2_DS_2_-VASc scores with a median of 4. No patient had a history of stent thrombosis. The median HAS-BLED score was 2 in both groups. A history of bleeding was present in 13.0% of patients in group 1 compared to 8.1% of patients in group 2 (*p* = 0.39). The main indication for anticoagulation with DOAC was atrial fibrillation/ atrial flutter in 93.8% vs. 88.7% in group 1 vs. group 2 (*p* = 0.19), respectively. Venous thromboembolism was the second most common reason and occurred significantly more often in group 2 than in group 1 (16.1% vs. 6.8%; *p* = 0.02). Approximately one quarter was treated for an acute coronary syndrome (*p* = 0.75).

**Table 1:** Patient characteristics at baseline.

Characteristic	Group 1	Group 2	*p*-value
	(*n* = 322)	(*n* = 62)	
Age—years	76.3 ± 8.3	74.9 ± 9.1	0.122
median (IQR)	78 (71–82)	76 (69–80)	
Female sex	88 (27.3)	17 (27.4)	>0.999
Body weight—kg^a^	83.0 ± 17.5	80.7 ± 18.3	0.154
median (IQR)	81 (70–93)	76 (69–88)	
Diabetes mellitus	101 (31.4)	19 (30.6)	>0.999
Hypertension	274 (85.1)	47 (75.8)	0.090
Heart failure	113 (35.1)	23 (37.1)	0.773
Hypercholesterolemia	171 (53.1)	33 (53.2)	1.000
Prior stroke or TIA	39 (12.1)	11 (17.7)	0.222
CHA_2_DS_2_-VASc score^b^	4.5 ± 1.4	4.4 ± 1.3	0.545
median (IQR)	4 (4–5)	4 (4–5)	
HAS-BLED score^c^	2.2 ± 0.9	2.1 ± 0.9	0.842
median (IQR)	2 (1–3)	2 (1–2)	
Prior myocardial infarction	88 (27.3)	18 (29.0)	0.759
Prior PCI	169 (52.5)	25 (40.3)	0.096
Prior stent thrombosis	0 (0.0)	0 (0.0)	n/a
Prior CABG	61 (18.9)	8 (12.9)	0.285
Prior bleeding	42 (13.0)	5 (8.1)	0.394
Current smoking	30 (9.3)	6 (9.7)	>0.999
Creatinine clearance—ml/min.^d^	70.9 ± 29.1	77.3 ± 39.3	0.469
median (IQR)	66 (52–85)	66 (51–92)	
Creatinine clearance 30 to <60 ml/min	100/280 (35.7)	21/60 (35.0)	>0.999
Creatinine clearance <30 ml/min	8/280 (2.9)	2/60 (3.3)	0.691
Serum creatinine ≥1.5 mg/dl	46 (14.3)	6 (9.7)	0.420
Hemoglobin—mg/dl	13.0 ± 1.7	13.0 ± 1.8	0.672
median (IQR)	13 (12–14)	13 (12–14)	
Platelet count—10^3^/*μ*l^e^	212.0 ± 72.3	215.0 ± 70.3	0.708
Median (IQR)	202 (172–239)	207 (183–257)	
Indication for DOAC^f^			
Atrial fibrillation or flutter	302 (93.8)	55 (88.7)	0.186
Venous thromboembolism	22 (6.8)	10 (16.1)	**0**.**023**
Intracardiac thrombus	5 (1.6)	0 (0.0)	>0.999
Indication for PCI			
Acute coronary syndrome	80 (24.8)	17 (27.4)	0.750
STEMI	5 (1.6)	2 (3.2)	0.315
NSTEMI	43 (13.4)	8 (12.9)	>0.999
Unstable angina	32 (9.9)	7 (11.3)	0.818
Chronic coronary syndrome	242 (75.2)	45 (72.6)	0.750

Values are presented as mean ± SD, median (IQR) or *n* (%). Bold values indicate statistical significance.

TIA, transient ischemic attack; PCI, percutaneous coronary intervention; DOAC, direct oral anticoagulation; CABG, coronary artery bypass graft; STEMI, ST elevation myocardial infarction; NSTEMI, non-ST elevation myocardial infarction.

^a^ Available in *n* = 340 cases, *n* = 280 in group 1 and *n* = 60 in group 2.

^b^ A higher CHA_2_DS_2_-VASc score indicates a higher risk of stroke.

^c^ A higher HAS-BLED score indicates a higher risk of major bleeding.

^d^ Creatinine clearance was calculated with the use of the Cockcroft–Gault equation and was available in *n* = 340 cases, *n* = 280 in group 1 and *n* = 60 in group 2.

^e^ Available in *n* = 355 cases, *n* = 298 in group 1 and *n* = 57 in group 2.

^f^ Patients could have more than one indication for DOAC.

### Procedural characteristics and peri-procedural antithrombotic therapy

Procedural characteristics are detailed in Table 2. The majority of procedures was performed via radial access. Patients predominantly presented with multivessel disease (group 1: 84% vs. group 2: 74%; *p* = 0.22). All patients received DES only, and an average of 1.6 stents were deployed per PCI, without differences between the two groups (*p* = 0.62). There were no differences in cumulative stent length (*p* = 0.33), rate of bifurcation stenting (*p* = 0.53), and overlapping stents (*p* = 0.84). However, group 2 received slightly larger stent diameters (3.2 ± 0.4 mm vs. 3.0 ± 0.4 mm, *p* = 0.014) and was more frequently stented in the left main stem than group 1 (7.9% vs. 2.5%, *p* = 0.019). The most frequently stented vessel in both groups was the left anterior descending artery (*p* = 0.98).

**Table 2:** Procedural Characteristics and Periprocedural Antithrombotic Therapy.

Characteristic	Group 1	Group 2	*p*-value
	(*n* = 322)	(*n* = 62)	
Access			
Radial	197 (61.2)	43 (69.4)	0.253
Femoral	158 (49.1)	27 (43.5)	0.488
Conversion	33 (10.3)	8 (12.9)	0.500
Size of sheath—Charrière^a^	6.1 ± 0.3	6.1 ± 0.3	0.847
Median (IQR)	6 (6–6)	6 (6–6)	
Angiographic finding			
One-vessel disease	60 (18.6)	16 (25.8)	0.223
Two-vessel disease	102 (31.7)	15 (24.2)	0.292
Three-vessel disease	160 (49.7)	31 (50.0)	>0.999
PCI			
Total stents deployed—no.	512	102	0.620
Stents per PCI	1.6 ± 0.8	1.6 ± 0.9	0.620
Median (IQR)	1 (1–2)	1 (1–2)	
Drug eluting stent	512/512 (100)	102/102 (100)	n/a
Bare metal stent	0/512 (0.0)	0/102 (0.0)	n/a
Stent length—mm^b^	25.6 ± 15.2	27.5 ± 16.7	0.333
Median (IQR)	20 (15–32)	22 (16–34)	** **
Stent diameter—mm^c^	3.0 ± 0.4	3.2 ± 0.4	**0**.**014**
Median (IQR)	3.0 (2.8–3.0)	3.0 (2.8–3.5)	** **
Bifurcation stenting	15 (4.7)	4 (6.5)	0.525
Stent overlap	44 (13.7)	9 (14.5)	0.842
Stented vessel			
LAD	230/512 (44.9)	43/102 (42.2)	0.980
LCX	98/512 (19.1)	14/102 (13.7)	0.655
RCA	149/512 (29.1)	35/102 (34.3)	0.495
Left main stem	13/512 (2.5)	7/102 (6.9)	**0**.**019**
Bypass graft	22/512 (4.3)	3/102 (2.9)	0.797
Medication			
Aspirin loading^d^	274 (85.1)	51 (82.3)	0.566
P2Y_12_ inhibitor loading^d^	211 (65.5)	47 (75.8)	0.140
Unfractionated heparin^e^	311 (96.6)	59 (95.2)	0.481
International units * 10^3^	8.3 ± 3.1	8.0 ± 2.9	0.227
Median (IQR)	8 (7–9)	7 (6–9)	
Glycoprotein IIb/IIIa inhibitors	1 (0.3)	1 (1.6)	0.297

Values are presented as mean ± SD, median (IQR) or *n* (%). Bold values indicate statistical significance.

PCI, percutaneous coronary intervention; LAD, left anterior descending artery; LCX, left circumflex artery; RCA, right coronary artery.

^a^ 1 Charrière (Ch) = 1/3 mm.

^b^ Calculated by adding lengths of all stents used.

^c^ Nominal diameter of the smallest stent, if >1 stent was used.

^d^ Patients who did not receive loading either had prior therapy with aspirin and/or P2Y_12_ inhibitor or loading was not reported, see main text for details.

^e^ See main text for details.

Aspirin loading in the catheter laboratory or preclinically was reported in 85.1% and 82.3% in group 1 and group 2, respectively. Patients in both groups received a median dose of 500 (100–500) mg intravenous aspirin (*p* = 0.253). 0.6% in group 1 and 1.6% in group 2 had prior aspirin therapy. Aspirin loading was not documented in 14.3% and 16.1% in group 1 and group 2, respectively (*p* = 0.70). Loading with a P2Y_12_ inhibitor was documented in 65.5% in group 1 and in 75.8% in group 2. In group 1 23.9% and in group 2 19.4% had prior therapy with a P2Y_12_ inhibitor. Documentation of loading with a P2Y_12_ inhibitor was missing in 10.6% in group 1 and in 4.8% in group 2 (*p* = 0.24). A total of five patients (group 1: 1.2% vs. group 2: 1.6%) received loading with the P2Y_12_ inhibitor ticagrelor and were switched to clopidogrel from the first post-interventional day, no patient received prasugrel, and all other patients received clopidogrel (Table 2). For 96.6% and 95.2% of procedures in group 1 and group 2, respectively, the application of periinterventional unfractionated heparin was documented (*p* = 0.48). In both groups, a glycoprotein IIb/IIIa inhibitor was used in only one PCI (*p* = 0.30).

### Post-procedural antithrombotic therapy

The post-procedural antithrombotic therapy is detailed in Table 3 and the Graphic Abstract. From the first post-procedural day, group 1 was treated with dual therapy (DOAC + clopidogrel) by definition. The most commonly used DOAC in group 1 was rivaroxaban (69.3%), followed by apixaban (27.3%), whereas dabigatran (2.2%) and edoxaban (1.2%) were rarely used. In group 2, by definition, all patients were treated with triple therapy (DOAC + clopidogrel + aspirin 100 mg/day) from the first post-procedural day. Aspirin therapy was continued for a mean of 1.5 ± 1.2 days after the procedure. The DOAC used most frequently in group 2 was rivaroxaban (56.5%), followed by apixaban (38.7%). Edoxaban (4.8%) was used infrequently and dabigatran (0.0%) was not used at all. Patients in group 1 were more frequently treated with rivaroxaban at a dose of 15 mg/day (instead of 20 mg/day) than those in group 2 (group 1: 67% vs. group 2: 51%; *p* = 0.03).

**Table 3:** Postprocedural antithrombotic therapy.

Antithrombotic therapy	Group 1^a^	Group 2^a^	*p*-value
	(*n* = 322)	(*n* = 62)	
Aspirin	0 (0.0)	62 (100)	n/a
Postprocedural aspirin therapy—days	0.0 ± 0.0	1.5 ± 1.2	n/a
Median (IQR)	0 (0–0)	1 (1–1)	** **
P2Y_12_ inhibitor			
Clopidogrel	322 (100)	62 (100)	n/a
DOAC			
Apixaban	88 (27.3)	24 (38.7)	0.092
5 mg/d	14 (4.3)	4 (6.5)	0.509
10 mg/d	74 (23.0)	20 (32.3)	0.146
Dabigatran	7 (2.2)	0 (0.0)	0.604
220 mg/d	6 (1.8)	0 (0.0)	0.595
300 mg/d	1 (0.3)	0 (0.0)	>0.999
Edoxaban	4 (1.2)	3 (4.8)	0.087
30 mg/d	1 (0.3)	0 (0.0)	>0.999
60 mg/d	3 (0.9)	3 (4.8)	0.056
Rivaroxaban	223 (69.3)	35 (56.5)	0.055
15 mg/d	214 (66.5)	32 (51.2)	**0**.**030**
20 mg/d	9 (2.8)	3 (4.8)	0.420

Values are presented as mean ± SD, median (IQR) or *n* (%). Bold values indicate statistical significance.

DOAC, direct oral anticoagulation.

^a^ Patients in group 1 received aspirin only at the day of procedure, while patients in group 2 received additional aspirin for ≥1 day.

### Endpoints

The mean duration of post-procedural hospitalization was 2.1 ± 2.5 and 2.2 ± 3.0 days in group 1 and group 2, respectively (*p* = 0.30). During this follow-up period, there were no myocardial infarctions, no stent thromboses, no strokes, no thromboembolic events, and no deaths. This was also confirmed for the subgroup of patients suffering from an ACS in both groups. One patient with chronic coronary syndrome in each group experienced a TIA (group 1: 0.3% vs. group 2: 1.6%; *p* = 0.29) (Table 4). TIMI major and minor bleeding occurred in 2.2% and 4.8%, ISTH major or clinically relevant nonmajor bleeding occurred in 4.0% and 8.1% and GUSTO severe or moderate bleeding was reported in 1.6% and 3.2% in group 1 and group 2, respectively (Table 4), all these differences did not reach statistical significance. Study endpoints are provided in Table 4 and the Graphic Abstract.

**Table 4:** Study Endpoints.

	Group 1	Group 2	*p*-value
	(*n* = 322)	(*n* = 62)	
Follow-up—days	2.1 ± 2.5	2.2 ± 3.0	0.305
Median (IQR)	1 (1–2)	1 (1–2)	
Ischemic events			
Myocardial infarction	0 (0.0)	0 (0.0)	n/a
Stent thrombosis	0 (0.0)	0 (0.0)	n/a
Stroke	0 (0.0)	0 (0.0)	n/a
TIA	1 (0.3)	1 (1.6)	0.297
Thromboembolic event	0 (0.0)	0 (0.0)	n/a
Death	0 (0.0)	0 (0.0)	n/a
Bleeding events			
TIMI major or minor bleeding	7 (2.2)	3 (4.8)	0.208
TIMI major bleeding	1 (0.3)	1 (1.6)	0.297
TIMI minor bleeding	6 (1.9)	2 (3.2)	0.621
TIMI bleeding requiring medical attention	10 (3.1)	3 (4.8)	0.449
ISTH major or clinically relevant nonmajor bleeding	13 (4.0)	5 (8.1)	0.186
ISTH major bleeding	9 (2.8)	4 (6.5)	0.239
ISTH clinically relevant nonmajor bleeding	4 (1.2)	1 (1.6)	0.587
GUSTO severe or moderate bleeding	5 (1.6)	2 (3.2)	0.315
GUSTO severe bleeding	0 (0.0)	0 (0.0)	n/a
GUSTO moderate bleeding	5 (1.6)	2 (3.2)	0.315

Values are presented as mean ± SD, median (IQR) or *n* (%). One patient per group had a TIA. Both patients were discharged one day after the intervention. There were no other ischemic events. Patients in group 2 had numerically more bleeding events, but these differences were not significant.

TIA, transient ischemic attack; TIMI, thrombolysis in myocardial infarction; ISTH, international society on thrombosis and haemostasis; GUSTO: global use of strategies to open occluded arteries.

### Subgroup analysis

A subgroup analysis was performed on a total of 125 patients who were hospitalized for at least two post-procedural days. This resulted in a follow-up time of 4.4 ± 3.4 days in group 1 (*n* = 100) and 4.0 ± 4.1 days in group 2 (*n* = 25). The subgroup analysis also revealed no ischemic events and no statistically significant differences in bleeding events between group 1 and group 2 (Table 5).

**Table 5:** Subgroup analysis of patients hospitalized for ≥2 days.

Outcome	Group 1	Group 2	*p*-value
	(*n* = 100)	(*n* = 25)	
Follow-up—days	4.4 ± 3.4	4.0 ± 4.1	0.062
Median (IQR)	3 (2–5)	2 (2–4)	0.062
Ischemic events			
Myocardial infarction	0 (0.0)	0 (0.0)	n/a
Stent thrombosis	0 (0.0)	0 (0.0)	n/a
Stroke	0 (0.0)	0 (0.0)	n/a
TIA	0 (0.0)	0 (0.0)	n/a
Thromboembolic event	0 (0.0)	0 (0.0)	n/a
Death	0 (0.0)	0 (0.0)	n/a
Bleeding events			
TIMI major or minor bleeding	7 (7.0)	2 (8.0)	1.000
TIMI major bleeding	1 (1.0)	1 (4.0)	0.361
TIMI minor bleeding	6 (6.0)	1 (4.0)	>0.999
TIMI bleeding requiring medical attention	8 (8.0)	2 (8.0)	>0.999
ISTH major or clinically relevant nonmajor bleeding	12 (8.0)	3 (12.0)	>0.999
ISTH major bleeding	9 (9.0)	3 (12.0)	0.705
ISTH clinically relevant nonmajor bleeding	3 (3.0)	0 (0.0)	>0.999
GUSTO severe or moderate bleeding	5 (5.0)	1 (4.0)	>0.999
GUSTO severe bleeding	0 (0.0)	0 (0.0)	n/a
GUSTO moderate bleeding	5 (5.0)	1 (4.0)	>0.999

Values are presented as mean ± SD, median (IQR) or *n* (%). In this subpopulation, no ischemic events occurred and differences in bleeding events were not significant.

TIA, transient ischemic attack; TIMI, thrombolysis in myocardial infarction; ISTH, International Society on Thrombosis and Haemostasis, GUSTO, global use of strategies to open occluded arteries.

## Discussion

In this retrospective single-center study of patients with long-term DOAC treatment who underwent PCI with stenting, immediate post-procedural initiation of dual therapy and discontinuation of aspirin, as compared to triple therapy, did not increase the rate of in-hospital ischemic events. Patients who received triple therapy for at least one post-procedural day tended to have more bleeding events, but this was not statistically significant.

Current European guidelines and a North American consensus statement recommend triple therapy for up to 1-week after PCI for most patients with an indication for anticoagulation (4, 5, 12, 13). A more precise duration within this 1-week period is not suggested due to lack of evidence. This is because randomized controlled trials of dual therapy (DOAC + clopidogrel) vs. triple therapy (DOAC/OAC + clopidogrel + aspirin) allowed randomization within 72 h (PIONEER AF-PCI), 120 h (RE-DUAL PCI), 14 days (AUGUSTUS), and 5 days (ENTRUST-AF PCI) after the index procedure, which allowed at least a short course of triple therapy to be administered even in the dual therapy group, most likely until randomization (7–10, 12). The median/ average time to randomization was 1-day (range 1–2)/1.62 ± 7.98 days in the PIONEER trial, 1-day (range 1–2)/1.6 ± 1.2 days in the RE-DUAL PCI trial, 6 days (range 3–10 days)/6.6 ± 4.19 days in the AUGUSTUS trial, and 1.9 (0.9–3.2) days/2.2 ± 1.4 days in the ENTRUST-AF PCI trial (7–10, 12). To address this gap in evidence and explore the important question, whether dual therapy can be started immediately after PCI and an exclusive peri-procedural aspirin application is safe with respect to ischemic events, the present analysis was performed. After the first publication on dual therapy using DOAC and clopidogrel, the majority of patients with an indication for anticoagulation undergoing PCI received dual therapy from the first post-procedural day in our center, resulting in an appropriate population to address this issue. Given the irreversible inhibition of the cyclooxygenase-1 enzyme by aspirin and the lifespan of platelets of 7–10 days, this approach appeared safe (22).

Our most important finding was that the rate of in-hospital ischemic events was indeed very low and not increased by discontinuation of aspirin, especially no acute stent thromboses occurred. Even in the subgroup of patients suffering from an ACS, discontinuation of aspirin appeared safe regarding ischemic events. This may be attributed to the long-lasting effect of aspirin and the short follow-up (mean ∼2 days, median 1 day). While the follow-up in the whole cohort was without doubt short and may have missed some events in the first week after PCI, it was sufficient to fill the gap of evidence described above. Moreover, the majority of stent thromboses occurs very early after PCI, so even a short follow-up appears relevant (23, 24). A subgroup analysis of patients with a mean follow-up of ∼4 days confirmed the low ischemic risk of very early dual therapy. There were no differences in established risk factors for stent thrombosis—such as prior stent thrombosis, diabetes mellitus, renal insufficiency, current smoking, baseline hemoglobin, multivessel disease, stent length, and type of presentation (NSTEMI/STEMI)—between the two groups (14). The very short follow-up period should be considered when interpreting our data, which could only assess acute stent thromboses within 24 h after stenting and to some extend subacute stent thromboses within few days. Subacute thromboses up to 1 month after stent implantation, late (1 month to 1 year) and very late thromboses (>1 year) could not be excluded on the basis of our data. These longer periods have been covered by the above-mentioned randomized trials of dual therapy.

It should be noted that our study, like the large randomized trials, was clearly underpowered to exclude differences in rare ischemic endpoints between dual and triple therapy, but it adds to the available evidence of the randomized trials that dual therapy has no excessive ischemic risk and supports the hypothesis that very early discontinuation of aspirin may be safe. Much larger trials with longer follow-up are needed to confirm our preliminary findings.

There were no significant differences in bleeding events between the two groups, but patients who received triple therapy for at least one post-procedural day numerically had a two-fold increased bleeding rate. This is in line with the available robust evidence that much more bleeding events occur while patients are receiving triple vs. dual therapy (3, 7–11). Use of the 20 mg dose of rivaroxaban was slightly more frequent in group 2, which may have increased bleeding risk under triple therapy. There was no relevant difference in other predisposing factors for bleeding events, reflected by the similar HAS-BLED scores and similar major and minor criteria for high bleeding risk as proposed by a consensus document from the Academic Research Consortium for High Bleeding Risk (16, 25).

To our knowledge, the present work—although it must be considered as hypothesis generating—is the first on exclusive peri-procedural aspirin application and immediate post-procedural initiation of dual therapy with DOAC and clopidogrel. This strategy may be attractive in clinical practice, since post-discharge prescription of aspirin and potential unintended prolonged aspirin intake can be avoided. Notably, about a quarter of patients treated with PCI at our center had an indication for anticoagulation, which is much more frequent than previously reported (1–3). This may be explained by the preferred referral of high-risk patients to a tertiary center, nevertheless it underlines the importance of this clinical scenario in real life.

### Limitations

There are a number of limitations to the present study. Due to its retrospective design, a possible lack of documentation of ischemic or bleeding events cannot be excluded, however this would have affected both analyzed groups in a similar way. Loading with a P2Y_12_ inhibitor was not documented in some cases in both groups. This is most likely a documentation error and not a missing application, since loading with a P2Y_12_ inhibitor is a highly respected clinical standard, which is triple-checked by nurses, interventional cardiologists and ward physicians in our clinic. However, we cannot exclude that loading has been indeed forgotten in a few cases. Missing documentation of P2Y_12_ inhibitor loading tended to be more frequent in group 1, so a potentially lower loading rate would rather strengthen than weaken our finding that early discontinuation of aspirin is safe. As discussed in detail above, due to low event rates, limited statistical power and short follow-up, findings should be interpreted with caution and must be considered as hypothesis generating. The majority of patients received either rivaroxaban or apixaban, while edoxaban or dabigatran were rarely used, so strictly speaking it remains unclear whether very early dual therapy is safe with all DOAC, although a class affect appears likely.

## Conclusion

In conclusion, about one out of four patients undergoing PCI has an indication for anticoagulation. Exclusive peri-procedural application of aspirin and initiation of dual therapy from the first post-procedural day appears safe regarding short-term ischemic events. A careful risk-benefit assessment should be performed and very early dual therapy might be considered in selected patients at low risk for ischemic events and/or high bleeding risk. However, larger studies with longer follow-up periods are needed to confirm these preliminary findings.

## Data Availability

Raw data may be shared upon reasonable request after approval of the indicated ethics committee. Requests to access the datasets should be directed to MH, marcel.halbach@uk-koeln.de.

## References

[1] Michniewicz E Mlodawska E Lopatowska P Tomaszuk-Kazberuk A Malyszko J. Patients with atrial fibrillation and coronary artery disease—double trouble. Adv Med Sci. (2018) 63(1):30–5. 10.1016/j.advms.2017.06.005 28818746

[2] Capodanno D Angiolillo DJ. Management of antiplatelet and anticoagulant therapy in patients with atrial fibrillation in the setting of acute coronary syndromes or percutaneous coronary interventions. Circ Cardiovasc Interv. (2014) 7(1):113–24. 10.1161/CIRCINTERVENTIONS.113.001150 24550531

[3] Capodanno D Huber K Mehran R Lip GYH Faxon DP Granger CB Management of antithrombotic therapy in atrial fibrillation patients undergoing PCI. J Am Coll Cardiol. (2019) 74(1):83–99. 10.1016/j.jacc.2019.05.016 31272556

[4] Collet J-P Thiele H Barbato E Barthélémy O Bauersachs J Bhatt DL 2020 ESC guidelines for the management of acute coronary syndromes in patients presenting without persistent ST-segment elevation. Eur Heart J. (2021) 42(14):1289–367. 10.1093/eurheartj/ehaa575 32860058

[5] Knuuti J Wijns W Saraste A Capodanno D Barbato E Funck-Brentano C 2019 ESC Guidelines for the diagnosis and management of chronic coronary syndromes. Eur Heart J. (2020) 41(3):407–477. 10.1093/eurheartj/ehz425 31504439

[6] van Rein N Heide-Jørgensen U Lijfering WM Dekkers OM Sørensen HT Cannegieter SC. Major bleeding rates in atrial fibrillation patients on single, dual, or triple antithrombotic therapy. Circulation. (2019) 139(6):775–86. 10.1161/CIRCULATIONAHA.118.036248 30586754

[7] Gibson CM Mehran R Bode C Halperin J Verheugt FW Wildgoose P Prevention of bleeding in patients with atrial fibrillation undergoing PCI. N Engl J Med. (2016) 375(25):2423–34. 10.1056/NEJMoa1611594 27959713

[8] Vranckx P Valgimigli M Eckardt L Tijssen J Lewalter T Gargiulo G Edoxaban-based versus vitamin K antagonist-based antithrombotic regimen after successful coronary stenting in patients with atrial fibrillation (ENTRUST-AF PCI): a randomised, open-label, phase 3b trial. Lancet. (2019) 394(10206):1335–43. 10.1016/S0140-6736(19)31872-0 31492505

[9] Cannon CP Bhatt DL Oldgren J Lip GYH Ellis SG Kimura T Dual antithrombotic therapy with dabigatran after PCI in atrial fibrillation. N Engl J Med. (2017) 377(16):1513–24. 10.1056/NEJMoa1708454 28844193

[10] Lopes RD Heizer G Aronson R Vora AN Massaro T Mehran R Antithrombotic therapy after acute coronary syndrome or PCI in atrial fibrillation. N Engl J Med. (2019) 380(16):1509–24. 10.1056/NEJMoa1817083 30883055

[11] Dewilde WJM Oirbans T Verheugt FWA Kelder JC de Smet BJGL Herrman J-P Use of clopidogrel with or without aspirin in patients taking oral anticoagulant therapy and undergoing percutaneous coronary intervention: an open-label, randomised, controlled trial. Lancet. (2013) 381(9872):1107–15. 10.1016/S0140-6736(12)62177-1 23415013

[12] Angiolillo DJ Bhatt DL Cannon CP Eikelboom JW Gibson CM Goodman SG Antithrombotic therapy in patients with atrial fibrillation treated with oral anticoagulation undergoing percutaneous coronary intervention. Circulation. (2021) 143(6):583–96. 10.1161/CIRCULATIONAHA.120.050438 33555916

[13] Hindricks G Potpara T Dagres N Arbelo E Bax JJ Blomström-Lundqvist C 2020 ESC guidelines for the diagnosis and management of atrial fibrillation developed in collaboration with the European Association for cardio-thoracic surgery (EACTS). Eur Heart J. (2021) 42(5):373–498. 10.1093/eurheartj/ehaa612 32860505

[14] Gori T Polimeni A Indolfi C Räber L Adriaenssens T Münzel T. Predictors of stent thrombosis and their implications for clinical practice. Nat Rev Cardiol. (2019) 16(4):243–56. 10.1038/s41569-018-0118-5 30518952

[15] Lip GYH Nieuwlaat R Pisters R Lane DA Crijns HJGM Andresen D Refining clinical risk stratification for predicting stroke and thromboembolism in atrial fibrillation using a novel risk factor-based approach: the euro heart survey on atrial fibrillation. Chest. (2010) 137(2):263–72. 10.1378/chest.09-1584 19762550

[16] Pisters R Lane DA Nieuwlaat R de Vos CB Crijns HJGM Lip GYH A novel user-friendly score (HAS-BLED) to assess 1-year risk of major bleeding in patients with atrial fibrillation: the euro heart survey. Chest. (2010) 138(5):1093–100. 10.1378/chest.10-0134 20299623

[17] investigators GUSTO. An international randomized trial comparing four thrombolytic strategies for acute myocardial infarction. N Engl J Med. (1993) 329(10):673–82. 10.1056/NEJM199309023291001 8204123

[18] Schulman S Kearon C. Subcommittee on control of anticoagulation of the scientific and standardization committee of the international society on thrombosis and haemostasis. Definition of major bleeding in clinical investigations of antihemostatic medicinal products in non-surgical patients. J Thromb Haemost. (2005) 3(4):692–4. 10.1111/j.1538-7836.2005.01204.x 15842354

[19] Kaatz S Ahmad D Spyropoulos AC Schulman S. Definition of clinically relevant non-major bleeding in studies of anticoagulants in atrial fibrillation and venous thromboembolic disease in non-surgical patients: communication from the SSC of the ISTH. J Thromb Haemostasis. (2015) 13(11):2119–26. 10.1111/jth.13140 26764429

[20] Chesebro JH Knatterud G Roberts R Borer J Cohen LS Dalen J Thrombolysis in myocardial infarction (TIMI) trial, phase I: a comparison between intravenous tissue plasminogen activator and intravenous streptokinase. Clinical findings through hospital discharge. Circulation. (1987) 76(1):142–54. 10.1161/01.CIR.76.1.142 3109764

[21] Mehran R Rao Sv Bhatt DL Gibson CM Caixeta A Eikelboom J Standardized bleeding definitions for cardiovascular clinical trials: a consensus report from the bleeding academic research consortium. Circulation. (2011) 123(23):2736–47. 10.1161/CIRCULATIONAHA.110.009449 21670242

[22] Funk CD Funk LB Kennedy ME Pong AS Fitzgerald GA. Human platelet/erythroleukemia cell prostaglandin G/H synthase: cDNA cloning, expression, and gene chromosomal assignment. FASEB J. (1991) 5(9):2304–12. 10.1096/fasebj.5.9.1907252 1907252

[23] Kuramitsu S Ohya M Shinozaki T Otake H Horie K Kawamoto H Risk factors and long-term clinical outcomes of second-generation drug-eluting stent thrombosis. Circ Cardiovasc Interv. (2019) 12(6):e007822. 10.1161/CIRCINTERVENTIONS.119.007822 31177822

[24] Singh K Rashid M So DY Glover CA Froeschl M Hibbert B Incidence, predictors, and clinical outcomes of early stent thrombosis in acute myocardial infarction patients treated with primary percutaneous coronary angioplasty (insights from the university of Ottawa heart institute STEMI registry). Catheter Cardiovasc Interv. (2018) 91(5):842–8. 10.1002/ccd.27215 28733995

[25] Urban P Mehran R Colleran R Angiolillo DJ Byrne RA Capodanno D Defining high bleeding risk in patients undergoing percutaneous coronary intervention. Circulation. (2019) 140(3):240–61. 10.1161/CIRCULATIONAHA.119.040167 31116032 PMC6636810

