# Supplementary material for: Early in-hospital discontinuation of aspirin on the first post-procedural day after percutaneous coronary stent implantation in patients on direct oral anticoagulation
Source: Front Cardiovasc Med. 2023 Dec 13;10:1265452. doi: 10.3389/fcvm.2023.1265452 (PMC10754416; doi:10.3389/fcvm.2023.1265452)
Supplement: Supplementary file 1 [file Datasheet1.docx]

# Supplemental Tables

## Table S1: Number of patients vs. PCIs

|  | **Patients (n)** | **PCIs (n)** |
| --- | --- | --- |
| **Total (n)** | 3613 | 4564 |
| **Indication for OAC (n)** | 871 | 1059 |
| **%/Total (%)** | 24.1 | 23.2 |
| **Group 1 (n)** | 284 | 322 |
| **Group 2 (n)** | 56 | 62 |

## Table S2: GUSTO Bleeding Definitions ^16^

| **Type of bleeding event** | **Definition of bleeding event** |
| --- | --- |
| Severe bleeding | Either an intracranial hemorrhage or bleeding that causes hemodynamic compromise and requires intervention. |
| Moderate bleeding | Bleeding that requires blood transfusion but does not result in hemodynamic compromise. |
| Mild bleeding | Bleeding that does not meet criteria for either severe or moderate bleeding. |

## Table S3: ISTH Bleeding Definitions ^17, 18^

| **Type of bleeding event** | **Definition of bleeding event** |
| --- | --- |
| Major bleeding | Clinically overt bleeding that is associated with:   1. Fatal bleeding, and/or 2. Symptomatic bleeding in a critical area or organ, such as intracranial, intraspinal, intraocular, retroperitoneal, intra-articular or pericardial, or intramuscular with compartment syndrome, and/or 3. Bleeding causing a fall in hemoglobin level of 2 g/dL or more, or leading to transfusion of 2 or more units of whole blood or red cells. |
| Clinically relevant nonmajor bleeding | Overt bleeding event not meeting the criteria for a major bleeding event, but associated with medical intervention, unscheduled contact with a physician, cessation of study drug treatment, or associated with discomfort for the subject such as pain or impairment of activities of daily life. Specific examples of clinically relevant non-major bleeding include the following:   1. Physician-guided medical or surgical treatment 2. Physician-guided change, interruption (omitting more than one dose) or discontinuation of the study drug. 3. Epistaxis if it lasts for more than 5 minutes, if it is repetitive, or leads to an intervention 4. Gingival bleeding if it occurs spontaneously, or if it lasts for more than 5 minutes 5. Haematuria if it is macroscopic, and either spontaneous or lasts for more than 24 hours after instrumentation 6. Macroscopic gastrointestinal haemorrhage: at least 1 episode of melena or haematemesis, if clinically apparent 7. Rectal blood loss, if more than a few spots 8. Haemoptysis 9. Multiple sources of bleeding 10. Surgery |
| Minimal bleeding | Other overt bleeding event that does not meet the criteria for major or clinically relevant nonmajor bleeding events. |

## Table S4: TIMI Bleeding Definitions ^19^

| **Type of bleeding event** | **Definition of bleeding event** |
| --- | --- |
| Major bleeding | Any intracranial bleeding (excluding microhemorrhages < 10 mm only evident on gradient-echo MRI)  Clinically overt sign of hemorrhage associated with a fall in hemoglobin level of 5 g/dL or more, or an absolute decrease in hematocrit of 15% or more  Fatal bleeding (bleeding that directly results in death within seven days) |
| Minor bleeding | Clinically overt sign of hemorrhage and fall in hemoglobin level of 3 – 5 g/dL, or an absolute decrease in hematocrit of 10 – 15 %. |
| Requiring medical attention | Any overt sign of hemorrhage that meets one of the following criteria and does not meet criteria for a major or minor bleeding event, as defined above:   1. Requiring intervention (medical practitioner-guided medical or surgical treatment to stop or treat bleeding, including temporarily or permanently discontinuing or changing the dose of medication or study drug) 2. Leading to or prolonging hospitalization 3. Prompting evaluation (leading to an unscheduled visit to a healthcare professional and diagnostic testing, either laboratory or imaging) |
| Minimal bleeding | Any clinically overt sign of hemorrhage associated with a fall in hemoglobin level of less than 3 g/dL or an absolute decrease in hematocrit of 9 % or less as well as any clinically overt sign of hemorrhage not fulfilling criteria for major and minor bleeding. |
